# Reliability and Validity of the Turkish Version of the Acute Stroke Management Questionnaire

**DOI:** 10.1002/brb3.70191

**Published:** 2024-12-22

**Authors:** Öznur Adadıoğlu, Bilgehan Atılgan Acar, Türkan Acar

**Affiliations:** ^1^ Internal Medicine Nursing Department Sakarya University, Faculty of Health Science Sakarya Türkiye; ^2^ Department of Neurology Sakarya University Faculty of Medicine Sakarya Türkiye

**Keywords:** acute stroke, health‐care professionals, reliability, validity

## Abstract

**Objective:**

The current research was carried out to test the validity and reliability of the Turkish version of the Acute Stroke Management Questionnaire (ASMaQ), developed to determine the acute stroke management awareness of health‐care professionals.

**Methods:**

This methodological study was performed in a training and research hospital. Data were collected using the “Participant Interview Form” and the Acute Stroke Management Questionnaire.

**Results:**

The Content Validity Index (CVI) of the Acute Stroke Management Questionnaire was found to be 0.91. As a result of exploratory factor analysis (EFA), it was revealed that the scale, which was adapted to Turkish, consisted of 3‐factor and 29 items, as in its original form. Confirmatory factor analysis confirmed the 3‐factor structure of the scale. The ASMaQ exhibited strong internal consistency (Cronbach's alpha 0.96) and test–retest reliability (0.879).

**Conclusion:**

The 29‐item and three‐dimensional structure of the Acute Stroke Management Questionnaire was revealed to be a valid and reliable measurement tool that could be utilized in evaluating the acute stroke management awareness of health‐care professionals in the Turkish language and culture.

## Graphical abstract

In this study, Figure 1 shows the scree plot of the acute stroke management questionnaire, and Figure 2 shows the path diagram of the CFA result of the acute stroke management questionnaire. The presence of 3 components with an eigenvalue above 1 indicates that the scale has a 3‐factor structure (Figure 1). The acute stroke management questionnaire confirmatory factor analysis diagram acquired from the confirmatory factor analysis is shown in Figure 2. Chi‐square = 1092.96, *df* = 371 *p*‐value = 0.00000, RMSEA = 0.072.

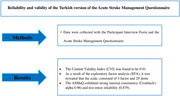

## 1 Introduction

In all societies, stroke is among the most important causes of disability and mortality in adults. Although there has been a 42% decrease in stroke incidence in high‐income countries in the last 40 years, it is remarkable that this rate has increased by 100% in low‐income countries (T.C. Sağlık Bakanlığı and Sağlık Hizmetleri Genel Müdürlüğü 2020). Stroke is among the most important health problems in Türkiye and is the second leading cause of death. Along with the gradual prolongation of life expectancy in Türkiye, a significant increase in stroke frequency is observed. It is predicted that the incidence of stroke will continue to increase in Türkiye in the coming decades (Ince, Necioglu, and Turkish Stroke Study Group).

There are numerous factors that can cause a delay in stroke treatment. Among these factors, the fact that stroke is not recognized as an emergency and inadequacies in its identification come to the forefront (Çakir and Öztürk 2017). Intervening in an acute stroke within hours is of vital importance. Hence, all health‐care professionals should have knowledge about stroke diagnosis and treatment approaches (Bayraktar et al. 2021). The competence of health‐care professionals in stroke care is a requirement to provide high‐quality patient care (Jarva et al. 2021). Sufficient knowledge and skills of health‐care professionals about stroke management will help reduce the burden of stroke on patients, their family members, and the health‐care system (Rababah, Al‐Hammouri, and AlNsour 2021). Several studies have demonstrated that health‐care professionals do not have sufficient knowledge about acute stroke, and diagnosis is established late, which causes delays in treatment (Abraham et al. 2017; Albart et al. 2022; Shire et al. 2017). The study by Albart et al. (2022) determined that health‐care professionals other than doctors had insufficient knowledge about stroke. A study from Dubai reported that the awareness of emergency personnel of the diagnosis and management of acute stroke was poor (Shire et al. 2017). Another study stated that the lack of awareness of health‐care professionals regarding stroke and the lack of coordination in the stroke care system were factors delaying acute stroke management (Abraham et al. 2017). There are many protocols and guidelines created for stroke management by the Ministry of Health in Türkiye (T.C. Sağlık Bakanlığı and Sağlık Hizmetleri Genel Müdürlüğü 2020; T.C. Sağlık Bakanlığı, Sağlık Araştırmaları Genel Müdürlüğü, and Sağlık Teknolojisi Değerlendirme Daire Başkanlığı 2017; Topcuoglu et al. 2020). Nevertheless, a limited number of studies conducted in Türkiye revealed that the acute stroke knowledge and awareness of health‐care professionals were not at the desired level (Altunışık and Tak 2021; Karadeniz and Yılmaz 2021; Yıldız 2018).

The lack of a comprehensive scale for the acute stroke management of health‐care professionals in Türkiye constituted the starting point of the current research. Therefore, the present study aimed to introduce the ASMaQ, which evaluates General Stroke Knowledge, Hyperacute Stroke Management, and Advanced Stroke Management, to the Turkish society. The literature review showed that the current study was the first to test the scale's validity in a different language. Since the scale has not yet been tested in different countries, it is thought that the results of the present research will contribute to international and national literature.

## 2 Materials and Methods

In this methodological study, the ASMaQ was adapted to Turkish society and culture. The study was conducted between May and July 2022 in a training and research hospital with a stroke center in Sakarya province, located in the west of Türkiye. In order to calculate the sample size, it was required for the questionnaire item number to be about five to ten times greater than the participant number, and in order to perform a factor analysis, it was required to include at least 100 individuals (Hair et al. 2019). Therefore, the study comprised 150 health‐care professionals for exploratory factor analysis (EFA) and 290 health‐care professionals for CFA who met the inclusion criteria and volunteered to take part. The participants who worked as health‐care professionals (specialist doctors, residents, nurses, health officers, and midwives) for at least 1 year and were on active duty were included in the study on a voluntary basis. For test–retest reliability, the scale was reapplied to 66 participants at an interval of 2 weeks.

### 2.1 Data Collection Tools

#### 2.1.1 Participant Interview Form

The form consisted of questions about the sociodemographic (age, gender, occupation, and working year) characteristics of the individuals.

#### 2.1.2 Acute Stroke Management Questionnaire

The ASMaQ was developed by Sim et al. in 2021. The questionnaire was designed to reveal the awareness of health‐care professionals of acute stroke management. The 29‐item scale includes the following three subscales:

General Stroke Knowledge (GSK): items 1, 2, 3, 4, 5, 6, 7, 8, 9, and 10
Hyperacute Stroke Management (HSM): items 11, 12, 13, 14, 15, 16, 17, 18, and 19
Advanced Stroke Management (ASM): items 20, 21, 22, 23, 24, 25, 26, 27, 28, and 29

The questionnaire is a five‐point Likert scale ranging from 1 to 5. Its 7 items (8–11, 17, 18, 29) are reverse coded. The ASMaQ has no cut‐off points. The maximum score that can be acquired from the scale is 145. A score close to 145 indicates that health‐care professionals have good acute stroke management knowledge. Cronbach's alpha value of the questionnaire was 0.82 (Sim et al. 2021). Measurement characteristics of the ASMaQ's Turkish version were evaluated by following the guidelines summarized in the COSMIN checklist (Mokkink et al. 2010).

### 2.2 Intercultural Adaptation

This process includes translation of the original form into Turkish, retranslation of the translated version into English, expert review, and preliminary application (Sousa and Rojjanasrirat 2011).

#### 2.2.1 Translation and Retranslation of the ASMaQ

Two independent translators translated the questionnaire into Turkish. The translators worked individually. The translations were combined and turned into a single form by the researchers. Then the questionnaire was retranslated into English by two different professional translators who were competent in both Turkish and English (Çapık, Gözüm, and Aksayan 2018). The documents were evaluated by taking into account the translations of the translators, and a single translation was obtained. The final form was compared with the original scale.

#### 2.2.2 Expert Review

In terms of content validity, the obtained Turkish version and the original English version were submitted to the opinion of ten specialists (4 neurology specialists, 1 neurology lecturer, 1 emergency medicine specialist, 1 emergency medicine lecturer, 3 internal medicine nursing lecturers) working in different specialties related to the subject. The expert panel comprised stroke patients and members who had experience and publications in research and questionnaire adaptation. The Davis technique was employed in the study of content validity. The experts were asked to grade the questionnaire items in terms of their relationship with the Turkish culture, comprehensibility, and extensity according to a four‐point scale (1. Not appropriate, 2. Item needs improvement to be appropriate, 3. Useful but needs little changes, and 4. Very appropriate) and offer a suggestion, if possible (Almanasreh, Moles, and Chen 2019). For all the 29 items, ten experts gave the answer, “The item is appropriate, and the item needs a mild review.” No expert gave a negative answer for the items. In line with expert opinions, changes were made to items 4 and 6. Item 4, “Stroke patients can present with limb numbness,” was modified to “Stroke may occur in patients with numbness of the arms and legs.” Item 6, “Acute stroke can present with a reduced level of consciousness,” was changed to “Acute stroke may manifest itself with impaired consciousness.” These changes were made to obtain appropriate items in terms of clarity and comprehensibility.

#### 2.2.3 Preliminary Application

For comprehensibility and cultural convenience of the questionnaire draft items, a preliminary study was conducted with ten health‐care professionals with the same characteristics as the sample group. Individuals who took part in the preliminary application were not included in the sample group. The participants found the questionnaire to be comprehensible and culturally appropriate and stated that there was no indistinct statement. Therefore, no change was needed.

#### 2.2.4 The Procedure

The data were collected by employing the face‐to‐face interview method once, based on the self‐reports of individuals. The interview lasted approximately 10 min. The questionnaire was reapplied to a total of 66 people from the entire sample who could be reached for the second time within an interval of 2 weeks. Health‐care professionals were asked to determine a nickname on the forms and write this nickname on the form in the test–retest application.

### 2.3 Ethical Considerations

Permission to use the ASMaQ was received from the corresponding author via e‐mail. The approval for the research was acquired from the Non‐Interventional Clinical Research Ethics Committee (approval number: 89–2022) of a state university. Prior to data collection, the participants were informed about the study's objective, and their verbal consent was received. The Declaration of Helsinki was compiled within the scope of the study.

### 2.4 Data Analysis

The data acquired from the research were analyzed with SPSS 22 (Statistical Package for Social Sciences) and LISREL 8.8 package software. In evaluating the study data, frequency distribution (number, percentage) for categorical variables and descriptive statistics (mean, standard deviation) for numerical variables were given. EFA and CFA were carried out to analyze the study's construct validity. Before applying factor analyses to test the suitability and adequacy of the size of the data set for analysis, the KMO > 0.80 and Bartlett's test of sphericity (*p* < 0.05) were used (Koyuncu and Kilic 2019). CFA was applied by employing the maximum likelihood estimation method and the fit indices of *χ* ^2^/SD, RMSEA, GFI, CFI, IFI, AGFI, and GFI (Xia and Yang 2019).

To determine the scale's reliability, Cronbach's alpha reliability coefficient, test–retest analysis, and item‐total score correlation analysis were performed. In the internal consistency analysis, Cronbach's alpha coefficient of 0.70 was accepted as sufficient (Lisawadi et al. 2019). In the item analysis, a positive correlation value of >+ 0.30 for each item was taken into consideration (Boateng et al. 2018). Pearson's correlation coefficient value of 0.70 was accepted as sufficient because of the test–retest (Akoglu 2018). Convergent validity was assessed by composite reliability (CR) and average variance extracted (AVE). The convergent validity criteria of the model were CR > AVE, with an AVE value being > 0.5 and a CR value being > 0.7 (Cheung et al. 2023). In order to ensure discriminant validity, according to the Fornell and Larcker criterion, the square roots of the AVE values must be higher than the correlation coefficients between the factors (Lim 2024).

## 3 Results

It was found that 58% of the participants were female, 24.1% were specialist doctors, 25.7% were residents, 40.7% were nurses, 5.2% were health officers, and 4.3% were midwives, while 45.2% worked for less than 5 years, 25.5% worked between 5 and 10 years, and 29.3% worked for more than 10 years. It was identified that the participants’ mean age was 32.58 ± 7.63.

### 3.1 Content Validity

Content validity was conducted using the opinions of ten experts. The CVI value was determined as 0.91 in the present study.

### 3.2 Construct Validity

**FIGURE 1:**
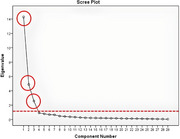
The presence of three components with an eigenvalue above 1 indicates that the scale has a 3‐factor structure.

The construct validity of the ASMaQ was assessed using factor analysis. According to the result of the KMO test (0.94), the sample size was determined to be sufficient for factor analysis. According to Bartlett's test, the scale items were highly correlated with each other (*χ* ^2^ = 5034.866; *p* = 0.000). The mentioned results demonstrated that the data were suitable for EFA. The varimax rotation method was employed in the factor analysis. As a result of the analysis, it was seen that the scale items were grouped under three factors. The presence of 3 components with an eigenvalue above 1 indicates that the scale has a 3‐factor structure. Scree plot also demonstrated that the scale had a 3‐factor structure (Figure 1).

Furthermore, it was also investigated whether the scale's factor structure corresponded to the original scale. It was observed that the scale comprised 3 factors as the original scale, and the factors of the items matched up with the original scale. The first factor included items 1, 2, 3, 4, 5, 6, 7, 8, 9, and 10. The item factor loadings were found to vary between 0.598 and 0.779, explaining 22.560% of the total variance. This factor was named “general stroke knowledge.” It was seen that the second factor consisted of items 11, 12, 13, 14, 15, 16, 17, 18, and 19. The item factor loadings were found to vary between 0.819 and 0.936, explaining 27.612% of the total variance. This factor was named “hyperacute stroke management.” The third factor comprised items 20, 21, 22, 23, 24, 25, 26, 27, 28, and 29. The item factor loadings were determined to be between 0.572 and 0.853, explaining 24.791% of the total variance. This factor was named “advanced stroke management” (Table 1).

**TABLE 1:** Results of the exploratory factor analysis for ASMaQ.

Items and subscales				Factor loading
**Factor I** **(General Stroke Knowledge)**				
9. In acute stroke, high blood pressure should be reduced to normal values.				0.598
7. The Glasgow Coma Scale (GCS) is a scale used to assess the level of consciousness.				0.732
10. Health‐care professionals should be provided with regular training on acute stroke management.				0.735
1. Acute confusion may be a sign of stroke.				0.766
5. Imbalance in walking may be a sign of stroke.				0.772
2. Hypoglycemia can mimic acute stroke.				0.774
3. Stroke can occur with visual disturbances.				0.779
8. Patients presenting with symptoms suggesting acute stroke should undergo a detailed neurological examination immediately.				0.779
4. Stroke may occur in patients with numbness of the arms and legs.				0.802
6. Acute stroke may manifest itself with impaired consciousness.				0.804
**Factor II (Hyperacute Stroke Management)**				
17. Coagulation profile should be screened before thrombolytic therapy.				0.819
12. All acute stroke patients should undergo a brain CT immediately.				0.848
18. All acute stroke patients should undergo a 12‐lead ECG before thrombolytic therapy.				0.871
15. Thrombolytic therapy is administered intravenously to break up clots.				0.897
19. Intracranial bleeding is a contraindication for thrombolytic therapy.				0.898
16. Thrombolytic therapy can be applied at the hospital where I work.				0.907
11. Stroke is a medical emergency for only 4.5 h from stroke onset.				0.930
13. All patients with suspected stroke should promptly consult the neurology team.				0.931
14. The sooner treatment begins, the better the outcome of acute stroke treatment is.				0.936
**Factor III (Advanced Stroke Management)**				
29. Strokes recognized on awakening are not suitable for thrombolytic therapy or mechanical thrombectomy.				0.572
27. Mechanical thrombectomy can be performed after thrombolytic therapy.				0.732
28. Thrombolytic therapy and mechanical thrombectomy can only be performed within the therapeutic time frame.				0.765
21. Do you have any knowledge about FAST (Face, Arm, Speech, and Time)?				0.771
25. Mechanical thrombectomy can be performed at the hospital where I work.				0.782
24. Mechanical thrombectomy is performed to eliminate the clot in acute stroke.				0.794
23. Do you have any knowledge about the mechanical thrombectomy treatment used for stroke?				0.795
22. How would you rate your knowledge about acute stroke management?				0.800
26. Acute stroke symptoms are potentially reversible with the administration of thrombolytic therapy or mechanical thrombectomy.				0.833
20. Can you detect the symptoms of acute stroke?				0.853
	**Factor I**	**Factor II**	**Factor III**	**Factors I–III**
Eigenvalue =	6.542	8.007	7.189	
Explained variance =	22.560	27.612	24.791	
Total explained variance =				74.963
Bartlett's test of sphericity =				5034.866
Kaiser–Meyer–Olkin value =				0.941

When the 29‐item scale was examined as a whole, it was seen that 3 factors explained 74.963% of the total variance, and the scale explained sufficient variance. The acquired values demonstrated that the scale was sufficient to explain the acute stroke management awareness of health‐care professionals. CFA was conducted to confirm the explained factor structure. The three‐dimensional structure of the ASMaQ was confirmed using confirmatory factor analysis (CFA). For the scale's construct validity, the goodness of fit statistics in CFA must be at the desired level. Upon examining the values of the fit criteria obtained as a result of CFA, it was found that the ratio of the most important fit value, *χ* ^2^, to the *df* value was 2.945, the RMSEA value was 0.072, the CFI value was 0.98, the GFI value was 0.94, the AGFI value was 0.92, the NNFI value was 0.98, the NFI value was 0.98, and the SRMR value was 0.047 (Table 2).

**TABLE 2:** CFA fit indices of the ASMaQ.

Fitness measurements	Good fit	Acceptable fit	Measurement value	Fit
*χ* ^2^/sd	0≤ *χ* ^2^ /*df*≤ 2	2≤ *χ* ^2^ /*df*≤ 3	2.945	Acceptable fit
RMSEA	0≤ RMSEA≤ 0.05	0.05≤ RMSEA≤ 0.08	0.072	Acceptable fit
SRMR	0 ≤ SRMR<0.05	0.05 ≤ SRMR ≤ 0.10	0.047	Good fit
NFI	0.95 ≤ NFI ≤ 1	0.90 ≤NFI < 0.95	0.98	Good fit
NNFI	0.97 ≤ NNFI ≤ 1	0.95 ≤NNFI < 0.97	0.98	Good fit
CFI	0.97 ≤ CFI ≤ 1	0.95 ≤CFI < 0.97	0.98	Good fit
GFI	0.95 ≤ GFI ≤ 1	0.90 ≤GFI < 0.95	0.94	Acceptable fit
AGFI	0.90 ≤ AGFI ≤ 1	0.85 ≤ AGFI < 0.90	0.92	Good fit

Abbreviations: AGFI: Adjusted goodness of fit index, ASMaQ: Acute Stroke Management Questionnaire, CFI: Comparative fit index, GFI: Goodness of fit index, NFI: Normalized fit index, NNFI: Non‐normalized fit index, RMSEA: root mean square error of approximation, SRMR: Standardized Root Mean Square Residual.

**FIGURE 2:**
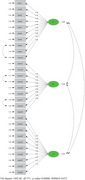
The acute stroke management questionnaire confirmatory factor analysis diagram acquired from the confirmatory factor analysis Chi‐Square = 1092.96 *df* = 371 *p*‐value = 0.00000 RMESA = 0.072.

Figure 2 shows the ASMaQ CFA diagram acquired from the CFA. The path diagram was examined, and the values found were observed to be suitable in terms of item‐factor fit.

### 3.3 Convergent Validity

The AVE and CR values are examined in this study. Analyses for convergent validity control found the AVE value to be 0.63 and the CR value to be 0.94 for the general stroke knowledge subscale, the AVE value to be 0.85 and the CR value to be 0.98 for the hyperacute stroke management subscale, and the AVE value to be 0.73 and the CR value to be 0.96 for the advanced stroke management subscale. CR values are higher than AVE values, and AVE values are higher than the critical value of 0.50. It can be said that convergent validity is provided by the values found.

### 3.4 Discriminant Validity

The analyses performed for discriminant validity control found the square root of the AVE to be 0.793 for the general stroke information subscale, 0.921 for the hyperacute stroke management subscale, and 0.854 for the advanced stroke management subscale. The correlation value between the general stroke information subscale and the hyperacute stroke management subscale was 0.409. The correlation value between the general stroke information subscale and the advanced stroke management subscale was 0.618. The correlation value between the hyperacute stroke management subscale and advanced stroke management subscale was 0.446. It is possible to state that with the values found, and the discriminant validity was ensured.

### 3.5 Reliability

Cronbach's alpha values of the ASMaQ are determined to be 0.942 for the “General Stroke Knowledge” factor, 0.981 for the “Hyperacute Stroke Management” factor, 0.96 for the “Advanced Stroke Management” factor, and 0.96 for the overall scale (Table 3). Item‐total correlations and Cronbach's alpha coefficients for each item were computed by employing the item elimination technique. As a result of the analysis, no item was removed since there was a significant correlation of 0.30 and above between the items and the scale total score. Furthermore, the alpha coefficient value of the scale was computed to determine to what extent and in what direction the items would affect the alpha coefficient if the item was deleted. Values indicate the internal consistency of the remaining variables if any variable is deleted. When any item is deleted, Cronbach's alpha values vary between 0.959 and 0.962. Upon examining the reliability values, no big change is observed. In other words, since it was observed that none of the items reduced the reliability value, no item was removed from the scale. The mean ASMaQ total score of health‐care professionals is 110.81 ± 19.81 (General Stroke Knowledge subscale 42.48 ± 6.65, Hyperacute Stroke Management subscale 32.77 ± 9.97, Advanced Stroke Management subscale 35.56 ± 7.79) (Table 3).

**TABLE 3:** The reliability on item‐total correlation and Cronbach's *α* if item deleted of the ASMaQ in Turkish version.

Items	Mean ± SD	Item‐total Correlation	Cronbach's *α*	Cronbach's *α* if item deleted
General Stroke Knowledge	42. 48 ± 6.65		0.942	
Item1		0.791		0.959
Item2		0.693		0.960
Item3		0.728		0.959
Item4		0.730		0.959
Item5		0.742		0.959
Item6		0.717		0.959
Item7		0.583		0.960
Item8		0.395		0.962
Item9		0,586		0,960
Item10		0.477		0.961
Hyperacute Stroke Management	32.77 ± 9.97		0.981	
Item11		0.769		0.959
Item12		0.793		0.959
Item13		0.741		0.959
Item14		0.746		0.959
Item15		0.759		0.959
Item16		0.752		0.959
Item17		0.717		0.959
Item18		0.724		0.959
Item19		0.780		0.959
Advanced Stroke Management	35.56 ± 7.79		0.960	
Item20		0.734		0.959
Item21		0.678		0.960
Item22		0.699		0.960
Item23		0.702		0.960
Item24		0.733		0.959
Item25		0.682		0.960
Item26		0.768		0.959
Item27		0.665		0.960
Item28		0.644		0.960
Item29		0.617		0.960
**Total ASMaQ**	110.81 ± 19.81		0.962	

The invariance of the scale was ensured by the test–retest performed with an interval. The scale was reapplied to 66 of the participants reached for the second time at an interval of two weeks. Pearson's correlation coefficient value was calculated as a result of the test–retest. The correlation coefficient value was 0.870 for General Stroke Knowledge, 0.885 for Hyperacute Stroke Management, 0.770 for Advanced Stroke Management, and 0.879 for the overall scale (Table 4).

**TABLE 4:** Test–retest results of ASMaQ (*n* = 66).

Scale/Subdimensions	Test		Retest		*r*
	Mean	SD	Mean	SD	
General Stroke Knowledge	4.40	0.64	4.14	0.64	0.870
Hyperacute Stroke Management	3.92	1.03	4.11	0.89	0.885
Advanced Stroke Management	3.77	0.78	3.78	0.87	0.770
Total ASMaQ	4.03	0.69	4.00	0.64	0.879

Abbreviation: *r*: Pearson correlation coefficient.

## 4 Discussion

The current research aimed to test the reliability and validity of the Turkish version of the ASMaQ. The literature review showed that this study was the first to test the scale's validity in a language different from the original language. Language, content, and construct validity were examined in this study since researchers need to be sure that the scale validly reflects the construct they plan to measure. In scale adaptation studies, first, language validity is investigated to determine the suitability of the inventory for the native language and culture. Afterward, the validity and reliability of the measurement tool are determined, and their cultural equivalences are compared. In the current study, the translation‐back translation method, which is frequently preferred by researchers, was employed for the language adaptation of the ASMaQ (Çapık, Gözüm, and Aksayan 2018).

Preliminary applications and examinations carried out to ensure language validity showed that the Turkish version of the ASMaQ was comprehensible and could be easily applied to the Turkish population. Content validity was evaluated after the language adaptation process.

While it is stressed in the literature that the CVI should be 0.80 and above, the value obtained in this study is observed to be at a very good level. The values acquired demonstrated that the Turkish version of the ASMaQ provided content validity and measured the targeted conceptual structure (Almanasreh, Moles, and Chen 2019). Since the CVI was found to be 0.91 in this study, it can be said that the ASMaQ measures the targeted conceptual structure.

The construct validity of the ASMaQ was tested with exploratory and CFA. The KMO test and Bartlett's test were carried out to evaluate whether the sample size was sufficient for factor analysis. For a sufficient sample size, the KMO value is expected to be more than 0.80, approaching 1 (Koyuncu and Kilic 2019). The fact that it was 0.94 in the present study shows that the sample is quite sufficient for factor analysis. Moreover, the fact that the result of Bartlett's test was determined to be significant demonstrates that the correlation between the variables is sufficient and the data set is suitable for the factor analysis.

It is considered sufficient if the percentage of factor loadings explaining the total variance is between 0.50 and 0.60, and it is stated that the higher the variance rates achieved are, the stronger the scale's factor structure is (Koyuncu and Kilic 2019). The three‐factor structure of the ASMaQ explains 74.96% of the total variance, demonstrating high construct validity. It can be said that the scale items are 0.40 and above (0.936–0.732) and have good distinctiveness, supporting the construct validity of all items (Taherdoost, Sahibuddin, and Jalaliyoon 2022).

It is suggested that the structure detected by EFA be examined using CFA. For the scale's construct validity, the goodness of fit statistics in the CFA must be at the desired level. The analysis showed that the scale fit in terms of compatibility values was good according to the SRMR, NFI, NNFI, CFI, and AGFI values and acceptable according to the *χ* ^2^/sd, RMSEA, and GFI values (Orcan 2018; Xia and Yang 2019). The study identified that the convergent validity of the measurement model was provided since the CR value was found to be above the threshold value of 0.70 and the AVE value was found to be above the threshold value of 0.50 (Cheung et al. 2023). Cronbach's alpha reliability coefficient method was employed to measure the internal consistency of the ASMaQ. If the detected value varies between 0.80 and 1.00, it is concluded that it has high reliability (Lisawadi et al. 2019). In this study, the square root of the mean explained variance value of each structure was greater than the relevant correlations of the structure, which shows that the discriminant validity had been ensured (Lim 2024).

Cronbach's alpha value was 0.820 (Sim et al. 2021). In the original scale and 0.96 in this study, which indicates that the Turkish version is highly reliable. Furthermore, since deleting any of the items in the scale does not increase Cronbach's alpha value of the scale, it was concluded that all items should be included in the scale (Hajjar 2018). Another measure of internal consistency is accepted as the item‐total correlation. For an item to be acceptable, the item‐total correlation coefficient should be positive and at least 0.30 (Boateng et al. 2018). In the current research, the item‐total correlation of the scale ranges from 0.395 to 0.793. Total score correlations are at a sufficient level for item analysis. The said findings demonstrate that the ASMaQ, which consists of 29 items, does not contain a problematic item in Turkish and has sufficient internal consistency.

In the present study, a test–retest analysis was conducted with the aim of determining the scale's consistency over time. The scale was applied to 66 health‐care professionals at an interval of two weeks using the test–retest technique. The acquired data were evaluated using Pearson's correlation analysis. The correlation coefficient calculated because of the analysis is expected to be above 0.70, and it is indicated that the higher the coefficient is, the more reliable the scores are (Akoglu 2018). The *r* value of the ASMaQ and its subscales was found to be high (*r *> 0.70) in the current study.

This result shows that the correlation between the scores received by the participants from the first test and the second test is high, and consistent measurements were obtained from the scale at different times, which shows that the tool is reliable.

## 5 Limitations of the Study

A limitation was that the study was carried out with health‐care professionals comprising specialist doctors, assistants, nurses, midwives, and health officers in only one hospital. The answers were based on the self‐report of individuals. Another limitation of the research is that the reliability and validity studies of the scale were not conducted in the languages of other countries, so it was only compared with the original scale. Another limitation was that the retest was performed two weeks after. It is of prime importance to consider these limitations when interpreting the outcomes of the present study. Despite these limitations, the current research contributes significantly to determining the stroke awareness of health‐care professionals, detecting incomplete and incorrect information, and the correct intervention in acute stroke cases, thereby improving the quality of care.

## 6 Conclusion

The ASMaQ is the first scale that measures the stroke management awareness of health‐care professionals in Türkiye. The fact that the scale has high psychometric properties shows that it is a valid and reliable scale that can determine the knowledge of health‐care professionals about acute stroke management in Türkiye. Health‐care professionals can apply the scale and determine their knowledge about acute stroke management. They can also use the data to plan training programs.

Future studies should further validate the Turkish version of the ASMaQ using a much larger sample of other health‐care professionals, such as home care personnel, physiotherapists, pharmacists, etc. Further studies should be conducted with a larger sample in community health centers, other types of hospitals, and other cities in Turkey. Moreover, in the studies to be conducted, longer ranges in the test–retest application may provide additional insight regarding the reliability of the measurement.

## Author Contributions

**Öznur Adadioğlu**: conceptualization, investigation, writing–original draft, writing–review and editing, visualization, validation, methodology, software, formal analysis, project administration, supervision, data curation. **Bilgehan Atılgan Acar**: conceptualization, investigation, writing–original draft, writing–review and editing, visualization, validation, methodology, software, formal analysis, data curation. **Türkan Acar**: conceptualization, investigation, writing–original draft, visualization, validation, methodology, software, formal analysis, data curation.

## Ethics Statement

The study was initiated after receiving the approval of Sakarya University, Faculty Medicine Clinical Research Ethics Committee (approval number: 04.04.2022‐89).

## Conflicts of Interest

The authors declare no conflicts of interest.

### Peer Review

The peer review history for this article is available at https://publons.com/publon/10.1002/brb3.70191.

## Data Availability

Research data are not shared.
