# Correction to “Reliability and Validity of the Turkish Version of the Acute Stroke Management Questionnaire”

**DOI:** 10.1002/brb3.70405

**Published:** 2025-04-09

1

Adadıoğlu, Ö., B. A. Acar, and T. Acar. 2024. “Reliability and Validity of the Turkish Version of the Acute Stroke Management Questionnaire.” *Brain and Behavior* 14, no. 12: e70191.

“Funding” section, the text “The authors received no specific funding for this work.” was incorrect. This should have read: “The study was supported by the TUBITAK ULAKBIM.”

We apologize for this error.

